# A Systematic Review of Meta-Analyses on Gene Polymorphisms and Gastric Cancer Risk

**DOI:** 10.2174/138920208785699544

**Published:** 2008-09

**Authors:** Francesco Gianfagna, Emma De Feo, Cornelia M van Duijn, Gualtiero Ricciardi, Stefania Boccia

**Affiliations:** 1 Institute of Hygiene, Università Cattolica del Sacro Cuore, Rome, Italy; 2 Department of Epidemiology & Biostatistics, Erasmus Medical Center, Rotterdam, The Netherlands

**Keywords:** Gastric cancer, meta-analysis, heterogeneity, polymorphism, population attributable risk.

## Abstract

**Background:**

Individual variations in gastric cancer risk have been associated in the last decade with specific variant alleles of different genes that are present in a significant proportion of the population. Polymorphisms may modify the effects of environmental exposures, and these gene-environment interactions could partly explain the high variation of gastric cancer incidence around the world. The aim of this report is to carry out a systematic review of the published meta-analyses of studies investigating the association between gene polymorphisms and gastric cancer risk, and describe their impact at population level. Priorities on the design of further primary studies are then provided.

**Methods:**

A structured bibliographic search on Medline and EMBASE databases has been performed to identify meta-analyses on genetic susceptibility to gastric cancer, without restriction criteria. We report the main results of the meta-analyses and we describe the subgroup analyses performed, focusing on the detection of statistical heterogeneity. We investigated publication bias by pooling the primary studies included in the meta-analyses, and we computed the population attributable risk (PAR) for each polymorphism.

**Results:**

Twelve meta-analyses and one pooled-analysis of community based genetic association studies were included, focusing on nine genes involved in inflammation (*IL-1β, IL-1RN, IL-8*), detoxification of carcinogens (*GSTs, CYP2E1*), folate metabolism (*MTHFR*), intercellular adhesion (*E-cadherin*) and cell cycle regulation (*p53*). According to their random-Odds Ratios, individuals carrying one of the *IL-1RN* *2*, IL-1β* -511T variant alleles or homozygotes for *MTHFR *677T are significantly at higher risk of gastric cancer than those with the wild type homozygote genotypes, showing high PARs. The main sources of heterogeneity in the meta-analyses were ethnicity, quality of the primary study, and selected environmental co-exposures. Effect modification by *Helicobacter pylori* infection for subjects carrying the unfavourable variant of *IL-1* polymorphisms and by low folate intake for individuals homozygotes for *MTHFR* 677T allele has been reported, while genes involved in the detoxification of carcinogens show synergistic interactions. Publication bias was observed (Egger test, *p *= 0.03).

**Discussion:**

The published meta-analyses included in our systematic review focused on polymorphisms having a small effect in increasing gastric cancer risk *per se.* Nevertheless, the risk increase by interacting with environmental exposures and in combination with additional unfavourable polymorphisms. Unfortunately meta-analyses are underpowered for many subgroup analyses, so additional primary studies performed on larger population and collecting data on environmental and genetic co-exposures are demanded.

## INTRODUCTION

Gastric cancer is the second leading cause of death from cancer worldwide, although mortality rates have been decreasing since several decades due to decreased incidence and improvement of survival [1]. Gastric carcinogenesis is a multistep and multifactorial process as a result of a complex interaction between environmental and genetic factors. Among the former, *Helicobacter pylori *infection, tobacco smoking, low fruit and vegetable intake, high meat and salt intake, and the lack of food refrigeration have been shown to be the major non-genetic determinants [2]. On the other side, a positive family history for gastric cancer is reported to be associated with the highest risk of gastric cancer [3]. In family studies, first degree relatives of patients with gastric cancer have two-to-three fold increase risk of gastric cancer not explained by familial clustering of *Helicobacter pylori *infection [4]. It has also been demonstrated that different genetic pathways lead to diffuse and intestinal subtypes of gastric cancer [5], confirming the functional role of genetic factors on cancer susceptibility. Beside the familial clustering, the individual genetic susceptibility to gastric cancer probably involves many genes [6], although their effects may only be small. However, the combination of even a few small effects could account for a sizeable population attributable fraction of gastric cancer. In the last decade the association between polymorphisms and gastric cancer has been investigated within community-based genetic association studies, aiming also to explore gene-gene and gene-environment interaction. Identifying the inherited genetic variants that modify the effect of well known nocive environmental exposures in gastric cancer risk could eventually lead to more effective primary intervention. This, however, is still an open issue [7].

According to the role of the genes whose polymorphisms have been studied in association with gastric cancer, we can grouped them into the following categories: genes involved in the protection of gastric mucosa against damaging agents, in inflammatory response, in detoxification of carcinogens, in synthesis and repair of DNA, in regulation of gene expression, in cell adhesion and in cell cycle [4,8]. Since the effect of each individual polymorphism could be small, association studies in genetic epidemiology benefit from large sample sizes. The majority of the published studies, however, are underpowered to detect a robust association. With the growing number of published papers, some authors attempted to quantitatively summarize the results of each individual polymorphism in association with gastric cancer by using the meta-analytical approach. This allows for enhancing the power of the association analysis by pooling data, and to identify potential causes of discrepancies among studies by performing sensitivity analyses while investigating data heterogeneity. Currently meta-analysis is the most cited study design in health sciences [9] and is widely accepted as the highest level of evidence in medicine. Initially adopted to summarize the results from clinical trials, meta-analyses were then widely applied to observational studies, including genetic epidemiology studies. The reliability of meta-analysis results, however, depends mainly on a rigorous methodology, on the quality of primary studies included and the availability for individual data collected from primary studies [10,11]. The latter aspect would allow to perform subgroup meta-analysis to explore gene-gene and gene-environment interaction, both suffering from a low power in individual studies. 

This systematic review of the published meta-analyses of observational studies investigating the relationship between gene polymorphisms and gastric cancer aims to: i) systematically review the role and the biological plausibility of each polymorphism in association with gastric cancer; ii) evaluate the attributable proportion of gastric cancer related to each polymorphismin the population ; iii) investigate the strengths and limitations of the published meta-analyses in order to suggest priorities for primary studies*.*

## METHODS

Identification of the meta-analyses of community-based association studies on polymorphisms and gastric cancer was carried out through a search of Medline and Embase, up to March 2008. The search strategy was carried out by combining the following terms: gastric, cancer, meta-analysis (with both synonymous and plural forms), as well as the truncated words ‘genetic*’, ‘allel*’ or ‘polymorphi*’, without any restriction on language. The references of retrieved papers was also examined to search for additional meta-analyses. The identified studies were screened by two authors independently (FG and EDF). The following topics were reported: biological role of each genetic polymorphism, gene expression at tissutal level, frequency of variant allele carriers in the population, overall results of the most appropriate observed genetic model from the meta-analysis reported as random effect Odds Ratio (OR) for gastric cancer and 95% confidence interval (CI), along with the *p* value for heterogeneity when available. Random effect model was used assuming heterogeneity between studies. Subgroup meta-analyses were reported when authors had actually performed, and observed changes in the statistical heterogeneity were discussed. 

The publication bias of the individual studies on gene polymorphisms and risk of gastric cancer was investigated by visual inspection of the funnel plot [12]. In order to have comparable data, the funnel plot was constructed by dividing the ORs of gastric cancer due to the unfavourable genotype from each individual study by the OR resulting from the corresponding published meta-analysis. The presence of publication bias was tested also with Begg and Mazumdar adjusted rank correlation test [13] and the Egger regression asymmetry test [14]. Lastly, we stratified the funnel plot according to the period of publication of the primary studies included in the meta-analysis. Accordingly, the first period of publication is defined as the period including primary studies published on the left fifty percentile of the time period spanning from the first paper published on that polymorphism to the last published one. 

Furthermore, in order to have a comprehensive view of the impact of each polymorphism on gastric cancer at population level, we computed their population attributable risk (PAR) among Caucasians and Asians. PAR for each polymorphism was computed as (OR-1)/OR * (number of exposed cases/total number of cases)] [15], using the ORs stratified for ethnicity and the pooled proportion of exposed cases for each ethnic subgroup both derived from the meta-analyses including the largest number of individuals. The 95% CI was computed as suggested by Natarajan *et al. *[16]. Statistical analyses were carried out using the STATA software package v.9.2 (Stata Corporation, College Station, Texas).

## POLYMORPHISMS AND GASTRIC CANCER RISK

Twelve meta-analyses and 1 meta- and pooled-analysis published since 2005 to 2008 were retrieved, focusing on 12 polymorphisms of 9 genes involved in inflammation (*IL-1A, IL-1B, IL-1RN, IL-8*), detoxification of carcinogens (*GSTM1, GSTT1, CYP2E1*), folate metabolism (*MTHFR*), intercellular adhesion (*E-cadherin*) and cell cycle regulation (*p53*) [17-29]. The main characteristics of each meta-analysis are reported in Fig. (**1**). The number of primary studies included in the meta-analyses ranged from 4 to 29, with a number of subjects included spanning from 2,500 to 10,000. Results of the meta-analysis including the largest number of individuals for each polymorphism are shown in Table **1**. The results of all the included meta-analyses are reported in the following paragraphs.

### Interleukin-1

Several genes involved in the inflammatory pathway have been investigated in relation with gastric cancer [8,30], in view of the immune response modulation in chronic gastritis, being one of the early phases in the development of intestinal gastric cancer type [31]. The interleukin (IL) genes are the most widely studied, especially the *IL-1*A, *IL-1*B and *IL-1RN*, contained in the *IL-1 *cluster*,* which encode for the proinflammatory cytokines IL-1α and IL-1β and their endogenous receptor antagonist IL-1RA, respectively [32].

IL-1β enhances the immune response with consequent free radical production, which may lead to lipid peroxidation and DNA damage, usually neutralized by efficient antioxidant defence system [33]. Furthermore, IL-1β inhibits gastric acid secretion, which could lead to increased production of gastrin, a cell growth factor involved in many processes, including neoplastic transformation [34]. *Helicobacter pylori* infection induces IL-1β production [35], and consequent hypochlorydria favours further colonization by pH-sensitive *Helicobacter pylori*, leading to the development of atrophic gastritis and adenocarcinoma [36]. It has been demonstrated that IL-1β overexpression can directly induce gastric atrophy and dysplasia both in presence or absence of *Helicobacter pylori* infection [37]. Three polymorphisms of the *IL1-B* gene have been observed to increase IL-1β expression: C**→**T transitions at both positions -511 and +3954 base pairs, and T**→**C at -31 base pairs from the transcriptional start. *IL-1B* -31C/T is a TATA-box polymorphism which was observed to affect DNA-protein interactions [38]. *IL-1B* -511T and *IL-1B* -31C are in near-complete linkage disequilibrium and the frequency of mutant alleles is 33% in Caucasians and about 50% in Asians. *IL-1B* +3954T frequency distribution ranges from 5-10% in Asiatic and Hispanic populations to 23% in Caucasians [22]. 

Interleukin-1 receptor antagonist (IL-1RA) is an important anti-inflammatory cytokine that can prevent the binding of *IL-1* to its cell-surface receptors, thus modulating its effects. A variable number tandem repeat (VNTR) polymorphism has been detected within intron 2 of the human interleukin-1 receptor antagonist gene (*IL-1RN*), consisting of repeats of an 86-bp sequence. Five allelic variants have been identified wherein the number of repeats varies from two (*2 allele) to six (long alleles, 3-6 repeats) [39]. Carriers of IL-1RN *2 variant allele produce high levels of *IL-1*β [36,40,41]. The *IL-1RN* VNTR region has 3 protein binding sites, suggesting a possible functional significance, although the mechanism underlying the association between the *IL-1RN* *2 allele and enhanced *IL-*1β expression is currently unknown. The frequency distribution of the IL1-RN *2 carrier status ranges from 6% in Asians to 27-30% among Caucasians and Hispanics [22]. 

**Fig. (1):**
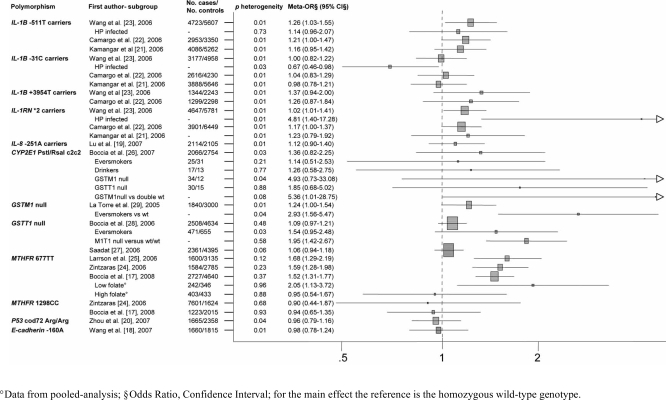
Description of the meta-analyses included in the systematic review.

The association between genetic variations within the *IL-1* gene cluster and gastric cancer was firstly investigated by El-Omar *et al.* in 2000 [38]. During 2006 three meta-analyses on *IL-1* gene cluster and gastric cancer were published almost at the same time. The largest one from Wang *et al.* included 39 primary studies, totalling for 7000 gastric cancer cases and 8500 controls [23], while 35 [21] and 25 primary studies [22] were included in remaining two studies (Fig. **1**). Results are discussed below, while results of the largest meta-analysis are reported in Table **1**. 

#### IL-1B -511T

In the meta-analysis of Camargo *et al.* [22], which included 14 studies, the dominant model resulted the most appropriate for -511T allele, according to the ORs derived by comparing the three genotypes [42]. Individuals carrying -511T variant allele have a borderline significantly increased risk of gastric cancer (OR = 1.21, 95%CI: 1.00-1.47) compared with CC homozygotes, however high level of heterogeneity resulted (*p* for heterogeneity <0.001). Subgroup analyses stratified by ethnicity, site, histological subtype, age and sex matching, quality of included studies and sample size, were carried out to explore potential sources of heterogeneity. High quality studies reported a meta-OR of 1.77 (95%CI: 1.49-2.09, *p* for heterogeneity = 0.81), while pooling only large sample sizes ( >400 subjects) studies an OR of 1.86 (95%CI: 1.50-2.31; *p* for heterogeneity = 0.72). Lastly, an OR of 1.66, (95%CI: 1.29-2.13; *p* for heterogeneity = 0.71) resulted from only non-cardia gastric cancer. Interestingly the increased risk for gastric cancer was confirmed in Caucasians, particularly among those with the intestinal subtype, while no association was found among Asians. 

These results were confirmed by the meta-analysis performed by Wang *et al.* [23], which included a larger number of cases and controls (Fig. **1**). An overall OR of 1.26 (95%CI: 1.03-1.55; *p* for heterogeneity <0.001) has been reported (Table **1**) for carriers of T allele *vs* CC. A meta-regression analysis suggests no effect of ethnicity, cancer histopathology*, Helicobacter pylori* infection and item related to methodological quality of included studies (*p*>0.1), although significantly higher risk of gastric cancer resulted for the intestinal subtype (OR = 1.76, 95%CI: 1.12-2.75) as well as for studies with characteristics related to low methodological quality (Table **1**). Interaction analysis with *Helicobacter pylori* infection (*p* value from meta-regression = 0.73) showed results similar to main analysis in general population (Table **1**). In subgroup analyses, a significantly higher risk of gastric cancer resulted for the intestinal subtype (OR = 1.76, 95%CI: 1.12-2.75, Table **1**) as well as for unmatched studies, those using a genotyping technique other than Polymerase Chain Reaction (PCR)- Restriction Fragment Length Polymorphisms (RFLP), and the papers published before 2003 (data not shown).

The meta-analysis performed by Kamangar *et al.* [21] reported a meta-ORs for CT heterozygotes of 1.07 (95%CI: 0.91-1.25; *p* for heterogeneity <0.001), and TT homozygotes of 1.16 (95%CI: 0.95-1.42; *p* for heterogeneity <0.001) compared with wild-type allele homozygotes (Fig. **1**). Subgroup analyses according to ethnicity, tumor location, hystological subtype, or possibility of bias due to genotyping provided no explanation of the heterogeneity and no significantly different results, despite confirming the trends shown in the other two meta-analyses [22,23]. 

#### IL-1B -31C

The meta-analysis of Camargo *et al. *[22], including 14 studies, suggested as appropriate the dominant genetic model, and showed a non-significantly increased risk of gastric cancer for those carrying C variant allele of *IL-1B* -31 compared with those wild-type homozygotes (OR = 1.04, 95%CI: 0.83-1.29; *p* for heterogeneity <0.001). Subgroup analyses (ethnicity, subsite, hystologic subtype, age and sex matching, quality of the included studies and sample size) failed to find out the heterogeneity source (data not shown). The meta-analysis of Wang *et al.* [23] which included 21 studies showed the absence of a significant association with gastric cancer (OR = 1.00, 95%CI: 0.82-1.22,* p* for heterogeneity <0.001). Meta-regression analyses showed that ethnicity, cancer histopathology, item related to methodological quality of included studies did not affect the systemic results (*p*>0.05, although for study quality p = 0.06), while *Helicobacter pylori *infection seems to have some impact in heterogeneity (p = 0.03), with -31C carriers having statistically significant decreased risk with respect to wild-type homozygotes among subgroups of HP infected (OR = 0.67; 95%CI: 0.46-0.98; Table **1**). Lastly, the meta-analysis of Kamangar *et al.* [21], which including 22 studies, did not report significant association neither for variant CC individuals (OR = 0.98, 95% CI: 0.78-1.21), nor for heterozygotes (OR = 0.99, 95% CI: 0.83-1.19; Fig. **1**) compared with wild-type homozygotes. Subgroup analyses (ethnicity, hystological subtype and cancer site) did not provide significant results (data not shown).

#### IL-1B +3954T

Two meta-analyses summarized the association between *IL-1B* +3954T polymorphism and gastric cancer (Fig. **1**) (Wang* et al. *[23], 10 studies; Camargo *et al. *[22], 8 studies). The small number of subjects carrying the variant TT genotype prevented the analysis of homozygous individuals, therefore a dominant genetic model was assumed [22]. According to both meta-analyses, individuals carrying +3954T mutant allele had an increased gastric cancer risk compared with +3954CC individuals, however not statistically significant (Camargo *et al.*: OR = 1.26, 95%CI: 0.87-1.24; *p* for heterogeneity <0.001; Wang *et al.*: OR = 1.37, 95%CI: 0.94-2.00; *p* for heterogeneity <0.001). None of the performed subgroup analyses explained the potential sources of heterogeneity, with heterogeneity decreasing only in subgroups including a limited number of studies and *p* value from meta-regression indicating a slight effect in heterogeneity for ethnicity (*p *= 0.04; non-significant ORs in subgroups). No interaction analysis was performed because of data unavailability.

#### IL-1RN *2

The association between IL-1RN*2 variant allele and gastric cancer has been summarized from Camargo *et al. *[22], Wang *et al. *[23], and Kamangar *et al.* [21] (Fig. **1**). The meta-analysis from Camargo *et al.* [22] suggested the recessive model as the most appropriate. Homozygotes for *2 allele have a non-significantly increased risk of gastric cancer (OR = 1.29, 95% CI: 0.82-2.02, *p* for heterogeneity <0.01) compared with carriers of long alleles (L), nevertheless a minor but slight statistically significant increase of gastric cancer risk resulted for carriers of the IL-1RN*2 allele (OR = 1.17, 95%CI: 1.00-1.37, *p* for heterogeneity <0.01) compared with the LL homozygotes. The subgroups analyses by ethnicity, subsite, hystologic subtype, age and sex matching, quality of included studies and sample sizes were performed for each of the genetic model used, showing decreased heterogenity among Asians and Hispanics (recessive model) and in subgroups of age and sex matched studies or with population-based controls (dominant model; data not shown). When considering only the intestinal subtypes of gastric cancer, both models provided increased risk, statistically significant only in the recessive model (OR = 2.26, 95%CI: 1.08-4.74). The meta-analysis of Wang *et al.* [23] reported a meta-OR of 1.20 (95%CI: 1.01-1.41; *p* for heterogeneity <0.01) for IL-1RN*2 carriers compared with *IL-1RN* homozygous wild-type individuals. Meta-regression including effect estimates and ethnicity, histopathology, age and sex matching, quality of included studies control source, publication time and *Helicobacter pylori *infection showed that possible source of heterogeneity are *Helicobacter pylori* infection (p<0.001), with ORs that became significant in both *Helicobacter pylori* infected (OR = 4.81, 95%CI: 1.40-17.28) and uninfected (OR = 1.83, 95%CI: 1.17-2.86). Genotyping methods also represents a major issue for this polymorphism, with only studies using PCR-RFLP methods confirming the main result (data not shown). Analyses from genotype contrasts by Kamangar *et al. *[21] showed ORs of 1.15 (95%CI: 0.96-1.38) and 1.23 (95%CI: 0.79-1.92) for *IL-1RN* *2 heterozygotes and for *2*2 homozygotes versus LL individuals, respectively. Subgroup analyses (ethnicity, hystological subtype and cancer site) did not provide significant results.

### Interleukin-8

Interleukin-8 (IL-8) is a member of the family of chemokines. Initially characterized for its leukocyte chemotactic activity it is mainly involved in the initiation and amplification of acute inflammatory reactions as well as the maintenance of chronic inflammatory processes [43]. The IL-8 cytokine is also involved in the gastric inflammatory response to *Helicobacter pylori* infection, through the recruitment and activation of immune cells and the stimulation of Reg protein expression which is a potent growth factor for gastric mucosal cells [44]. In addition it has been described that IL-8 has tumorigenic and proangiogenic properties [45]. The gene coding for IL-8 exhibits several functional polymorphisms, fifteen of them have been characterized [46]. Among these polymorphisms the presence of IL-8 -251 T/A in the transcription start site exerts a great influence on IL-8 production and an increased transcriptional activity of the IL-8 promoter was confirmed in an in-vitro assay [47, 48]. The frequency of the mutant allele -251A varied significantly among different ethnic groups, ranging from 38-51% in Europeans [49,50] and 30-42% in Asians [51,52]. 

A recently published meta-analysis on IL-8 -251A and gastric cancer included 8 case-control studies [19] (Fig. **1**). The combined OR was not significant for the individuals carrying the -251A allele compared with the homozygous wild-type genotype (OR = 1.12, 95%CI: 0.90-1.40;* p* for heterogeneity = 0.003). The heterogeneity decreased when one study was excluded from the analysis, resulting in a significantly higher risk for gastric cancer among carriers of the variant allele (fixed effect OR = 1.21, 95% CI: 1.06-1.39; *p* for heterogeneity =0.05). 

### Cytochrome P450 2E1

Cytochrome P450 2E1 (CYP2E1), a member of the cytochrome P-450 superfamily, is a naturally ethanol-inducible Phase I enzyme. It is mainly involved in the metabolic activation of low molecular weight compounds such as N-nitrosamines as well as in alcohol metabolism [53,54]. N-nitrosamines are formed endogenously in the stomach and are present in different environmental factors including tobacco smoke and some dietary compounds [55]. *CYP2E1* gene is expressed nearly ubiquitously [56]. Two point mutations in the 5’-flanking region (PstI, RsaI), which are in close linkage disequilibrium, are known to alter the gene transcriptional activity [53]. The resulting c2 variant allele (7% of Caucasians, 36% of Asians) [57,58] is associated with a higher protein production. The association between *CYP2E1 *polymorphisms and gastric cancer was firstly investigated by Kato *et al.* in 1995 [59]. The only one meta-analysis on *CYP2E1* *Pst*I/*Rsa*I and gastric cancer [26] included 13 studies published before 2005 (Fig. **1**). The meta-analysis (Table **1**) showed an overall OR of gastric cancer risk of 0.97 (95%CI: 0.79-1.18; *p* for heterogeneity = 0.01) for c2 variant allele carriers, and 1.36 (95%CI: 0.82-2.25; *p* for heterogeneity = 0.03) for c2 homozygotes compared with wild-type c1 homozygotes. Stratifying the results for ethnicity and quality score, a decreased heterogeneity in Caucasians (*p* for heterogeneity = 0.53 and 0.46 for c2 carriers and homozygotes, respectively) and high quality studies (*p* for heterogeneity = 0.08 for both models) was observed (Table **1**). High quality studies among Asians showed a significant increased risk of gastric cancer both among c2 heterozygotes and c2 homozygotes (*p* for heterogeneity = 0.71 and 0.38). Authors of the meta-analysis performed some gene-gene and gene-environment interaction meta-analysis by using individual level data provided by some authors of the studies included. No significant association was detected from gene-environment (smoking, alcohol) interaction meta-analysis, despite the biological plausibility, while an OR of 5.36 (95%CI: 1.01-28.47) for gastric cancer appeared for *CYP2E1 *c2 homozygotes with *GSTM1* null genotype, compared with individuals carrying both wild-type genotypes. In these interaction analyses the heterogeneity decreased, until a minimum value (*p* for heterogeneity = 0.58) in the gene-gene interaction analysis (double homozygotes contrasts).

### Glutathione S-Transferase

Glutathione S-tranferase (GST) is a family of genes, mainly involved in cell protection against electrophiles, including several environmental carcinogens, as well as endogenous products of oxidative stress [60]. These phase II enzymes bind glutathione, a nucleophilic tripeptide, to a wide spectrum of carcinogens, facilitating their detoxification [8]. Three major *GST* subfamilies are widely expressed in humans: *GSTM* (μ), *GSTT* (θ) and *GSTP* (π) with overlapping substrate specificities [61,62].* GSTM1* and *GSTT1 genes *exhibit homozygous deletion (null genotype) polymorphisms. Individuals carrying one of these variants have no enzyme activity, and thus are more susceptible to carcinogens such as benzo[α]pyrene-7,8-diol epoxide, the activated form of benzo[α]pyrene, and smaller reactive hydrocarbons, such as ethylene oxide and diepoxybutane [60,63], which could lead to environmentally-induced cancer susceptibility [64].

#### GSTM1 null

Glutathione S transferase M1 (*GSTM1*) is mainly expressed in liver, brain and stomach. The *GSTM1* null genotype is found in 10-60% of individuals ranging from 50% in Caucasians and Asians to 25% in Africans [29]. The association between *GSTM1* null genotype and gastric cancer was firstly investigated by Strange *et al.* in 1991 [65]. Two meta-analyses summarizing the results of individual studies are published and reported in Table **1**. 

The first meta-analysis evaluating the association between *GSTM1* status and gastric cancer included 15 primary studies in English language and was published in 2005 [29]. The meta-OR was 1.24 (95%CI: 1.00-1.54; *p* for heterogeneity <0.01) (Fig. **1**). The heterogeneity slightly decreased after stratifying by ethnicity (Asians, *p* for heterogeneity = 0.02), source of controls (hospital, *p* for heterogeneity = 0.06) and study power (>80% for OR=2.0 and α=0.05, *p* for heterogeneity = 0.12) (Table **1**). Caucasians showed a significantly increased risk (OR = 1.22, 95% CI: 1.04-1.43,) while studies with at least 80% power provided the lowest estimate (OR = 1.05, 95% CI: 0.87-1.28). As for gene-environmental analysis, the meta-analysis showed an OR of 2.93 (95%CI: 1.56-5.47; *p* for heterogeneity = 0.04) for ever-smokers with* GSTM1* null compared to never-smokers with *GSTM1* wild type genotype (Fig. **1** and Table **1**). Data on other types of interacting carcinogens, such as *Helicobacter pylori* infection and dietary compounds, were not available to perform any subgroup meta-analyses.

#### GSTT1 null

Glutathione S transferase T1 (*GSTT1) *is mainly expressed along the human gastrointestinal tract. The null genotype of *GSTT1* is present in 13-31% among Caucasians and 36-55% among Asians. The association between *GSTT1* null genotype and gastric cancer was firstly investigated by Deakin *et al.* in 1996 [66]. Two meta-analyses exploring this association, were contemporarily published in 2006 (Fig. **1**) [27,28]. The meta-analysis of Saadat [27] included 16 studies and reported an OR of 1.06 (95%CI: 0.94-1.19; *p* for heterogeneity > 0.05) for the association between *GSTT1* null genotype and gastric cancer. The meta-analysis of Boccia *et al*. [28] included 18 articles published in all languages and showed similar results (OR = 1.09, 95%CI: 0.97-1.21; *p* for heterogeneity = 0.48). According to Saadat [27], among the stratified analyses carried out for ethnicity, source of controls, sample size and smoking status, the subgroup of Caucasian studies showed a statistically significant increased risk of gastric cancer (OR = 1.27, 95%CI: 1.03-1.57), also confirmed by Boccia *et al.* (*p* for heterogeneity = 0.87) [28]. 

Both meta-analyses reported the absence of statistically significant association after restricting the analysis to the source of control or studies’ power, however Boccia *et al.* [28] showed a statistically significant association after restricting the analysis to high quality studies (OR = 1.23, 95%CI: 1.04-1.45; *p* for heterogeneity = 0.41). As for gene-environment interaction analysis, Saadat [27] reported an OR of 1.27 (95% CI: 0.94-1.72; *p>*005) for *GSTT1* null individuals compared to those with the normal genotype ever smokers. The identical subgroup analysis was performed by Boccia *et al.* [28], with the addition of data from two studies providing individual data, and results showed an OR of 1.54 (CI 95%: 0.95-2.48;* p* for heterogeneity = 0. 03) [28]. As for gene-gene interaction analysis, data on *GSTT1* and *GSTM1 *genotypes were combined in the two meta-analyses, using as reference group those carrying both homozygous wild genotypes. Saadat [27] extracted data from 4 studies and showed a significantly increased risk for individuals with the combined presence of *GSTM1* and *GSTT1* null genotypes if compared to those with both wild-type variant (OR = 2.08; 95% CI: 1.42-3.10). A similar result (OR = 1.95, 95% CI: 1.42-2.67; *p* for heterogeneity = 0.58) was shown by Boccia *et al.* [28], by pooling additional data obtained asking to the authors (total 7 studies). Saadat described an additive effect for *GSTT1* and *GSTM1* genotypes, taking into account the effects of each of the functional polymorphisms on gastric cancer risk [27]. Only few studies collected data on *Helicobacter pylori* infection and food consumption, in a way that was not enough to perform any subgroup meta-analyses.

### Methylenetetrahydrofolate Reductase

Methylenetetrahydrofolate reductase (MTHFR) is a key enzyme in folate metabolic pathway that irreversibly catalyzes the conversion of 5,10-methylenetetrahydrofolate to 5-methyltetrahydrofolate, the primary circulating form of folate and a cosubstrate for homocysteine methylation to methionine. Folic acid, a form of the water-soluble Vitamin B9, is a precursor of the metabolic pathway leading to DNA methylation which functions as a regulatory mechanism of gene expression. In addition folate plays an important role in transferring single-carbon methyl units during the synthesis of DNA and RNA and it is also involved in DNA repair. Folate intake is provided by diet and serum folate levels could be impaired by alcohol drinking, smoking habits and altered activity of several enzymes, such as MTHFR, serine hydroxymethyltransferase, thymidylate synthase and methionine synthase [67-69]. *MTHFR* is expressed ubiquitously and two functional polymorphisms, C677T and A1298C, have been identified [70]. Frequencies of individuals carrying the 677T allele are around 50% in Asians, 44% in Caucasians and 23% in African-Americans. The less frequent 1298C allele is present in 40% of Caucasians, 30% of Asians and 30% of African-Americans. Heterozygotes (CT) and homozygotes (TT) for C677T mutant allele have respectively 65% and 30% of the enzyme activity compared with individuals with the wild-type genotype, while 1298CC homozygotes have nearly 60% of the normal enzyme activity [70,71]. Individuals with the TT genotype for *MTFR* 677 have significantly lower plasma folate levels than those with the wild-type genotype, while for the *MTFR* 1298 variant the evidence is inconsistent [72]. Low folate levels might induce uracil misincorporation into DNA, which could lead to chromosomal breaks and mutations, and DNA hypomethylation, which results in altered gene expression and DNA conformation [73].

#### MTHFR 677T

The association between *MTHFR polymorphisms *and gastric cancer was firstly investigated by Shen *et al.* in 2001 [74]. In the last two years, three meta-analyses and one pooled analysis were published on *MTHFR* polymorphisms and gastric cancer [17,24,25]. According to the meta-analysis of Larrson *et al.* [25], which included 9 studies, individuals with the *MTHFR* 677TT genotype showed a higher risk of gastric cardia adenocarcinoma (OR = 1.90;95%CI: 1.38-2.60) and gastric cancer (OR = 1.68;95%CI: 1.29-2.19) compared with those wild-type homozygotes [25]. No significant heterogeneity was detected among the studies (*p *for heterogeneity = 0.29 and 0.12, respectively). The meta-analysis published by Zintzaras [24], including 8 studies (1584 cases and 2785 controls), reported similar results (data not shown). The least heterogeneity resulted for the recessive model, with the TT individuals having an increased gastric cancer risk compared with carriers of the C allele (OR = 1.47, 95% CI: 1.26-1.73; *p* for heterogeneity = 0.66). Stratification by gastric cancer site failed to decrease heterogeneity, while for East Asian studies a very low heterogeneity was observed (*p* for heterogeneity = 0.96 in the recessive model). The meta-analysis by Boccia *et al. *[17] which included 16 studies produced an overall OR of 1.52 (95% CI: 1.31-1.77; *p* for heterogeneity = 0.37) for gastric cancer and *MTHFR* TT genotype compared to the 677 CC. The subgroup analysis are reported on Table **1**. Since no data for interaction analyses were available to perform subgroup meta-analyses according to folate, alcohol and smoking, a pooled-analysis was carried out by Boccia *et al. *[17]. Data on 1540 gastric cancer cases and 2577 controls, and 1146 cases and 1549 controls were pooled for *C677T* and *A1298C*, respectively. Overall, the pooled analysis showed that *MTHFR* 677 TT individuals have an OR of 1.49 (95%CI: 1.14-1.95; *p* for heterogeneity = 0.06) for gastric cancer, thus confirming previous meta-analyses based on an unadjusted estimates. Sensitivity analyses based on ethnicity and gastric cancer site provided similar results, with a lower heterogeneity among East Asians (*p *for heterogeneity = 0.34). When results from the pooled analysis of four studies on *C677T* were stratified according to folate levels, results showed an increased risk among individuals with low levels (OR = 2.05; 95% CI: 1.13-3.72;* p* for heterogeneity = 0.96) respect to those with high folate levels (OR = 0.95; 95% CI: 0.54-1.67 *p *for heterogeneity = 0.86; *p *value among the two strata = 0.06). No interaction was detected from the stratified analyses according to alcohol and smoking habits, with decreased heterogeneity only in the unexposed subgroups (*p* for heterogeneity = 0.84 and 0.49; Table **1**).

#### MTHFR 1298C

Absence of statistically significant association of 1298C variant allele and gastric cancer was reported in all genetic models, from Zintzaras (4 studies, all from East-Asiatic populations) [24] and Boccia *et al.* (7 studies) (Table **1**) [17]. The subgroup analyses according to ethnicity and tumour site performed by Boccia *et al. *[17] provided similar results, with no evidence of heterogeneity. Identically, the meta-analysis by Zintzaras [24] showed absence of association between *MTHFR *A1298C polymorphism and gastric cancer even when restricting the analysis to studies conducted among East-Asians or studies whose controls were in Hardy-Weinberg equilibrium. 

### p53

The *p*53 tumor suppressor gene is one of the most commonly mutated genes in all types of human cancer and encodes a transcription factor involved in cell cycle regulation. The *p*53 acts as a tumor suppressor gene by inducing cell cycle arrest or apoptosis and requires loss of function mutations for cancer development. Even if *p*53 gene is highly polymorphic, with at least 13 single nucleotide polymorphisms described [75], the *p*53 exon4 Arg72Pro polymorphism is the only whose role has been extensively studied in relation to gastric cancer. A polymorphism in this codon, which consists in a single base pair change of either arginine (Arg) or proline (Pro), has been suggested to modulate *p*53-dependent apoptosis and modify sensitivity to chemotherapeutic agents [76,77]. The alterations in exon 4 of the *p53* gene in gastric cancer were firstly investigated by Shepherd *et al.* in 2000 [77], who reported the following genotype frequencies: Arg/Arg (54%) Arg/Pro (33%) Pro/Pro (14%). The most intriguing aspect of the initial study is that the genotype of the codon 72 polymorphic site varied significantly with race (*p* for heterogeneity = 0.0001) as follows: 64% of whites had the Arg/Arg genotype compared with 24% of blacks [77].

The association between *p53 *codon 72 polymorphism and gastric cancer was firstly investigated by Hiyama *et al.* in 2002 [78]. A meta-analysis on *p*53 exon4 Arg72Pro polymorphism and gastric cancer including 12 case-control studies has been recently published (Fig. **1** and Table **2**) [20]. The combined results showed no significant difference in genotype distribution between gastric cancer cases and controls, with all models presenting heterogeneity and the recessive model giving the highest OR (1.21, 95%CI: 0.92-1.58; *p* for heterogeneity = 0.01). Heterogeneity was tested by subgroup analyses on ethnicity, tumour location, stage, Lauren’s classification and histological type. A significantly lower frequency of the Arg/Arg genotype in gastric cancer cases compared with controls was reported among Asians (OR for Arg/Arg versus carriers of the mutant allele = 0.84, 95%CI: 0.72-0.99), while not among Caucasians. Considering cases-only analyses performed on Asiatic case-control studies, a significantly higher frequency of variant homozygous genotype was found for gastric cardia cancer with respect to other locations (OR = 3.20, 95%CI: 1.46-7.01), and for advanced stages (III/IV) respect to those with early stages (I/II) (OR = 1.48, 95%CI: 1.01, 2.16; Table **1**). Data on other potential carcinogens such *Helicobacter pylori* infection and diet were insufficient to perform any subgroup meta-analyses.

### E-cadherin

The *E*-cadherin gene encodes a transmembrane glycoprotein, mainly involved in the establishment and maintanance of intercellular adhesion, cell polarity and the normal architecture of epithelial tissues [79] as well as in cell signaling in conjunction with cytoplasmic catenin proteins [18]. E-cadherin glycoprotein, as a calcium-dependent intercellular adhesion molecule, is localized on the surfaces of epithelial cells in regions of cell-cell contact, commonly known as adherens junctions [80]. The loss of the adhesive function of E-cadherin is a critical step in tumour development and progression mediating the transition to an invasive phenotype in human epithelial cancers [81]. Li *et al. *[81] identified a C/A single nucleotide polymorphism at -160 base pairs from the transcriptional start site of the *E-cadherin* gene promoter, showing that the A allele decreased the transcriptional efficiency by 68% if compared to the wild-type C allele, probably due to a stronger transcriptional factor binding activity of the C allele than the variant allele A. Therefore the -160 C/A polymorphism might alter E-cadherin expression, increasing susceptibility to epithelial cancers. The frequency of the mutant allele -160A varied significantly among different ethnic groups and geographic areas, ranging from 43.4-23.3% of Europeans and 0-61% in Asians [18]. 

The association between *E-cadherin *polymorphism and gastric cancer was firstly investigated by Wu *et al.* in 2002 [82]. A recently published meta-analysis on *E*-cadherin polymorphism and gastric cancer [18], including 9 case-control studies, was conducted to explore the association between gastric cancer and the -160 C/A polymorphism (Fig. **1**). The combined OR was not significant for the -160A allele carriers compared with the homozygous wild-type genotype (OR = 0.98, 95%CI: 0.79-1.16;* p* for heterogeneity = 0.01). The heterogeneity substantially decreased when Asian and European were analysed separately, resulting in a significantly higher risk for gastric cancer among carriers of the variant A allele among Caucasians (OR = 1.45, 95% CI: 1.06-1.97;* p* for heterogeneity = 0.29), and a decreased risk among Asians (OR = 0.81, 95% CI: 0.67-0.99;* p* for heterogeneity = 0.27; Table **1**). 

## SOURCES OF HETEROGENEITY

An important aim of meta-analyses is to assess the extent to which different studies provide similar or dissimilar results, which is usually assessed by statistical tests such as *Q* or *I*^2^ statistics [83], and to explain the sources. Statistical heterogeneity usually reflects variability in the results of different studies on the same question, which might not always represent a bias. Genetic association could, in fact, have different strength in different populations and different settings. Nevertheless, some authors listed potential reasons for inconsistency in results of genetic associations [84]. Among them, true variation of underlying association between populations, allelic associations that might vary between study setting, effect modification by other genetic variants or environmental risk factors, misclassification of the outcome, population stratification, and finally variation in power between studies. The present systematic review shows that in the published meta-analyses on polymorphisms and gastric cancer the potential sources of heterogeneity was investigated, and decreased heterogeneity among homogeneous ethnic groups and among individuals exposed to the same environmental risk factor resulted in many cases.

When results were stratified for ethnicity, heterogeneity strongly decreases for* IL-1* -511T polymorphisms [22], *IL-1 *+3954T [22,23], *E* cadherin -160A [18], *GSTT1* null [28] and *MTHFR 677T *in the pooled analysis [17], as possibly for +3954T [23]. Wide differences in allele frequencies have been reported for the mentioned polymorphisms among ethnic groups. Study characteristics as age/sex matching, source of controls, genotyping methods, are the second most studied potential source of heterogeneity. Quality scoring systems of the primary studies included in a meta-analysis were recently suggested by some authors, aiming to provide a tool for distinguishing between high an low quality studies, with most of them taking into account the previously listed study characteristics. Four meta-analyses [22,23,26,28] included in this review used a quality scale system and results show that stratification for quality score decreased heterogeneity for *IL-1* -31, *IL-1 *-511 and *CYP2E1 *PstI/RsaI. Furthermore, the studies scored as of high quality reported stronger effect estimates than those of low quality in 3 [22,26,28] out of 4 meta-analyses (Table **1**). 

Subgroup analyses according to environmental co-exposures showed a decreased statistical heterogeneity, as expected. The final effect of a functional genetic variant on a certain disease is expected to change in presence of its environmental substrate, so that the effect estimate might be extremely heterogeneous among studies if this aspect is not taken into account. In the reviewed meta-analyses, stratified analyses according to environmental exposures biologically related to the polymorphisms showed a decreased heterogeneity in almost all the instances (Table **1**). Lastly, interaction between two polymorphisms might be an additional source of heterogeneity, and this was investigated in 3 meta-analyses [26-28] on metabolic genes (Table **1**). The subgroup analyses exploring gene-gene interaction were performed by comparing the effect of one polymorphism after stratifying for a second genetic risk factor, or by comparing homozygotes for both the unfavourable variants with double wild-type homozygotes. In both instances the heterogeneity decreased and the ORs increased, although reaching statistically significant results only when comparing opposite homozygotes [26-28]. Carcinogens are usually detoxified by several enzymes involved in the same pathway, so that the contemporarily lack of two or more enzyme functions could exponentially increase the effect of the carcinogens on disease risk. The identified synergistic interaction among *GST*s null genotypes as well as the relationship between *GSTM1* and *CYP2E1 *unfavourable variants suggest that a wide portion of gastric cancer could be attributable to the inheritance of both unfavourable polymorphisms. Unluckily, these are the only reported gene-gene interaction meta-analyses, which were clearly limited from the few amount of original gene-gene interaction data from the individual studies. As example, though 17 of 18 published studies collected data on both *GSTM1* and *GSTT1* genotypes, only 5 authors reported case-control data according the two gene variants, thus limiting the power of subgroup meta-analysis. In this sense, large pooled analysis on gene-gene interaction and ad-hoc multiple genotyping conducted within consortia are strongly desirable.

## PUBLICATION BIAS 

Publication bias may be seen as a type of selection bias afflicting the scientific literature, stemming from that evidence that studies showing ‘significant’ results are more likely to be published than those presenting ‘negative’ results. The most reasonable explanations for this phenomenon is that authors may fail to write ‘negative’ papers and submit them to journals, as results are reviewed less favourably, or because editors simply don’t want to publish negative results. Additionally, papers reporting ‘positive’ results are more frequently published on international journals while ‘negative’ results are more easily published on local journals [85]. 

**Fig. (2):**
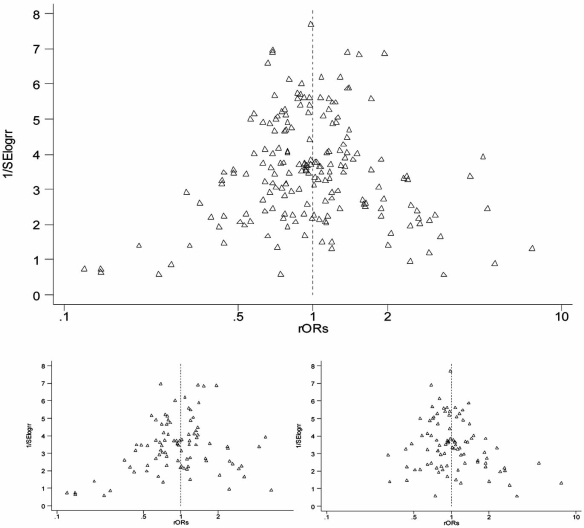
Funnel plot of pooled primary studies included in meta-analyses, before (bottom left) and after (bottom right) stratification according to the period of publication of the primary studies included in the meta-analysis (The first period of publication is defined as the period including primary studies published on the left fifty percentile of the time period spanning from the first paper published on that polymorphism to the last published one).

An accurate bibliographic search that foresee the retrieval of local literature can help minimizing this bias, however ascertaining the extent of publication bias is difficult, and typically we have no idea to what extent unpublished data distorts the literature. In almost all the included meta-analyses, publication bias was evaluated by visual inspection of funnel plot and/or with any asymmetry test, with observed asymmetry only in limited cases and of limited extent. However, asymmetry tests have limited power when the number of included studies is small. In this review we aimed to identify the presence and the source of publication bias, and a funnel plot was derived pooling all the primary data included in the meta-analyses, using the ratio of the study OR for the unfavourable polymorphism and the OR of the corresponding meta-analysis. This computation allows to have comparable values for different studies datasets included in the meta-analyses. A slight asymmetry resulted by visual inspection (Fig. **2**) and was confirmed by Egger (*p *= 0.029.) and Begg tests (*p *= 0.005). We also aimed to explore if the publication period affected the publication bias, and results show that the small outlier studies reporting statistically significant results were mainly published as first, while smaller studies which appear in the right side of the plot are the recently published ones (Fig. **2**, bottom left and right, respectively). A time-dependent asymmetry was confirmed by Egger and Begg tests, with *p* values of 0.002 and 0.008 in the subgroup of papers published during the second period of time, and 0.14 and 0.72 in the first period, respectively. Publication bias could also occur in the subgroup analyses, when collected data are reported by the authors in the full-text or indicated by the indexers in record field of bibliographic databases only if they are interesting. However, this issue is solved in the meta-analyses directly asking authors of the published papers to provide individual data [22,26,28].

## POWER

The lack of statistically significant results could be firstly due to the low power of some meta-analysis, which depends on the number of available studies and their results. *MTHFR 677T* variant allele seems to confer an increased risk of 50%, so the power to detect this association could reached by including even a few number of studies, while statistical significance for polymorphisms whose expected OR is 1.20 clearly needs a higher number of primary studies. In contrast the slight association between *IL*-1 polymorphisms and gastric cancer risk has been detected because many primary studies have been published on this issue. 

A comprehensive bibliographic search should be performed to provide a precise summary estimate given the existing studies on the same topic, avoiding, at the same time, selection bias. This is particularly true for* s*ubgroup analyses, which can be performed o the basis of data which could be collected and/or reported by few studies. In order to perform subgroup meta-analyses to explore gene-gene and gene-environment interaction with a greater power and avoid selection bias, contacting the authors of the published paper to ask for the unreported data or the entire dataset is highly recommended. Meta-analyses on *CYP2E1* and *GSTT1* genes obtained data on environmental and genetic co-exposures from the authors of the primary studies, and provided more precise estimates on *GSTT1* and smoking interaction [28], and *CYP2E1* and *GSTs* interaction [26]. The pooled analysis of *MTHFR* C677T and A1298C used the original data from around 50% of the published primary studies, however results did not differ from those of the meta-analysis of all published reports*. *Additional to the meta-analysis, however, the pooled analysis of *MTHFR* reported results of gene-environment interaction by folate, alcohol and smoking status, showing that *MTHFR* 677T carriers with a low folate status are at particularly increased risk of gastric cancer respect with those carrying the same genotype but with a high-folate status. 

## POLYMORPHISMS IMPACT

Overall, by summarizing the results of the published meta-analyses of the association between SNPs and gastric cancer, a statistically significant increased risk was reported for 3 polymorphisms, namely the *IL-1B* -511T and *IL-1RN* *2 variant allele (carriers status), and *MTHFR* 677T (homozygous status). A borderline significant increased risk was also shown for *GSTM1 *null variant. Few subgroups meta-analyses were performed due to absence of published data in the primary studies. Among them, however, some demonstrated a strongly increased risk for gastric cancer among those carrying the unfavourable variant of *IL-* who are contemporarily *Helicobacter pylori* positive [23], those contemporarily carrying *GSTM1* null and *CYP2E1* PstI/RsaI homozygous variant [26], and those contemporarily *GSTM1* null and *GSTT1* null [27,28]. Lastly, the subgroup meta-analysis on the pooled report of *MTHFR* C677T and gastric cancer, that was published together with a meta-analysis on the same research question, demonstrated that the risk of gastric cancer among those carrying the homozygous variant genotype actually increases only when the folate intake was low [17]. 

The actual impact of polymorphisms on gastric cancer risk in the population depends on both the strength of association and on the frequencies of variant alleles, which vary among ethnic groups. Calculating PAR for each polymorphism among Asians and Caucasians will provide the proportion of gastric cancer cases attributable to the presence of the variant genotype, as shown in Table **2**. From our computation, the most impacting polymorphisms are *E-cadherin* -160A and *IL-1*-511T (which might account for around 20% among Caucasians), *MTHFR* 677T variant and *GSTM1* null (both 10% in Asians and Caucasians), *IL-1RN* *2 and *IL-8* -251A (10% in Caucasians and Asians, respectively). The ORs and allele frequencies used for computing the PAR, however, are taken from population of different geographic areas, although the same ethnic group, which could bias the results. As such, further data are needed to obtain a reliable estimate of the PAR, including data concerning gene-gene or gene-environment interactions, in order to identify population subgroups for which the impact of a certain polymorphism might be particularly high (e.g., *MTHFR* 677T variant among those with a low dietary intake of folate).

## CONCLUSIONS

The integration of genome-based knowledge into healthcare has the potential to improve primary and secondary prevention [86]. Among the greatest promises of the genomic medicine is that the unravelling of the genetic origins of common diseases will lead to individualized medicine, in which the prevention and treatment strategies are personalized on the basis of the results of predictive genetic tests. Findings from meta-analyses of genetic association studies have the potential to provide a comprehensive view of the impact of genetic risk factors in disease aetiology, especially when exploring gene-environment interaction. The availability of gene-environment interaction data on gastric cancer, however, is currently limited. As such, primary well powered studies collecting data on environmental related co-exposures are strongly requested, since this would eventually lead to the identification of population subgroups at higher risk of gastric cancer because of a concomitant environmental co-exposure. Findings from the meta- and pooled analyses on genetic polymorphisms and risk of gastric cancer show an increased risk for individuals carrying *MTHFR* 677T allele when the folate status was low and *IL-1RN* *2 allele when *Helicobacter pylori* positive. At the moment the potential public health impact for both gene tests is limited, however, because of the limited amount of data on which results are based on. As for *MTHFR* gene, a proper evaluation of the clinical utility of *MTHFR* *C677T* testing for identifying gastric cancer susceptibility among populations with folate deficiency, as well as the introduction of specific folate supplementation (*versus* no folate supplementation) are therefore warranted. Identically, the evaluation of screening programs for *Helicobacter pylori* infection could be targeted according to the genetic make up of individuals, but this require a more extensive evaluation.

## Figures and Tables

**Table:** 

	*IL-1B *-511T Carriers [23]			*IL-1B *-31C Carriers [23]			*IL-1B *+3954T Carriers [23]			IL-1RN *2 Carriers [23]			*IL-8* -251A [19]			*p53 *codon 72 Arg/Arg [20]		
	*^*p** het.	OR	95% CI	*p* het.	OR	95% CI	*p* het.	OR	95% CI	*p* het.	OR	95% CI	*p* het.	OR	95% CI	*p* het.	OR	95% CI
Overall	<0.01	1.26	1.03-1.55	<0.01	1.00	0.82-1.22	<0.01	1.37	0.94-2.00	<0.01	1.20	1.01-1.41	0.03	1.12	0.90-1.40	0.04	0.96	0.79-1.16
Caucasian		1.42	0.97-2.06		1.10	0.81-1.50		1.15	0.84-1.57		1.30	1.09-1.54					1.32	0.89-1.97
Asian		1.16	0.92-1.46		0.92	0.71-1.18		1.73	0.59-5.05		1.09	0.78-1.52					0.84	0.72-0.99
High quality		1.07	0.80-1.42		0.98	0.73-1.30		1.67	0.84-3.32		1.13	0.86-1.49						
Population controls		1.13	0.94-1.36		0.96	0.78-1.19		1.15	0.97-1.36		1.21	1.00-1.45						
Published>2003		1.00	0.89-1.13		1.01	0.84-1.22		1.74	1.03-2.94		1.17	0.92-1.48						
Intestinal histotype		1.76	1.12-2.75		1.12	0.85-1.47		0.97	0.70-1.35		1.72	0.92-3.21				0.04§	1.10	0.64-1.89
Cardia																0.03§	0.91	0.53-1.55
Advanced																0.35§	1.48	1.01-2.16
Poor diferentiated																<0.01§	2.25	0.21-23.84
Interactions																		
*H. pylori* infected		1.41	0.96-2.07		0.67	0.46-0.98					4.81	1.4-17.28						

**Table:** 

	*CYP2E1 *PstI/RsaI c2c2 [26]			*GSTM1 *Null [29]			*GSTT1 *Null [28]			*MTHFR *677TT [17]			*MTHFR *1298CC [17]			*E-cadherin* -160A [18]		
	*p* het.	OR	95% CI	*p* het.	OR	95% CI	*p* het.	OR	95% CI	*p* het.	OR	95% CI	*p* het.	OR	95% CI	*p* het.	OR	95% CI
Overall	0.03	1.36	0.82-2.25	<0.01	1.24	1.00-1.54	0.48	1.09	0.97-1.21	0.37	1.52	1.31-1.77	0.93	0.94	0.65-1.35	0.01	0.98	0.78-1.24
Caucasian	0.46	0.42	0.05-3.85		1.22	1.04-1.43	0.87	1.27	1.03-1.56	<0.1	1.34	0.90-1.99		-	-	0.29	1.45	1.06-1.97
Asian	0.02	1.44	0.85-2.42		1.19	0.81-1.75	0.33	1.02	0.89-1.18	<0.1	1.64	1.36-1.97	<0.1	0.81	0.43-1.51	0.27	0.81	0.67-0.99
High quality	0.08	2.14	0.96-4.74				0.41	1.23	1.04-1.45									
Population controls				0.02	0.95	0.90-1.73	0.29	1.09	0.92-1.29									
Power >80% RR=2				0.12	1.05	0.87-1.28	0.27	1.08	1.24-0.47									
Genot. Methods				<0.01	1.20	0.97-1.47												
Cardia										<0.1	1.51	1.11-2.05	<0.1	0.99	0.43-2.28			
Pooled analysis										0.06	1.49	1.14-1.95	0.50	0.90	0.69-1.34			
Interactions																		
Eversmokers	0.21	1.14	0.51-2.53		2.93°	1.56-5.47	0.03	1.54	0.95-2.48									
Alcohol consumers	0.77	1.26	0.58-2.75															
*GSTT1* null	0.88	1.85	0.68-5.02															
*GSTM1* null	0.04	4.93	0.73-33.08				0.58°	1.95	1.42-2.67									
		5.36°	1.01-28.47															

§ Cases-only, category *vs* opposite category.

° Individuals exposed to both risk factors *vs* double unexposed: *GSTM1* null eversmokers *vs GSTM1* wild-type neversmokers; *GSTM1* and *GSTT1* null *vs GSTM1* and *GSTT1* wild-type; *CYP2E1* c2c2 and *GSTM1* null *vs CYP2E1* and *GSTM1* wild-type;

^ p for heterogeneity from Q-test when reported from the meta-analysis.

**Table 2:** Population Attributable Risk (PAR)° of Gastric Cancer Related to the Studied Polymorphisms

Polymorphism	Asians		Caucasians	
	PAR %	95% CI§	PAR %	95% CI
*IL-1B* -511T carrier status	10.2	(-9.4, 26.2)	18.3	(-5.2, 36.4)
*IL-1B* -31C carrier status	-6.8	(-37.6, 15.4)	5.3	(-19.3, 23.6)
*IL-1B* +3954T carrier status	6.7	(-24.1, 20.7)	5.3	(-11.4, 18.5)
*IL-1RN* *2 carrier status	1.2	(-6.8, 7.4)	10.8	(2.3, 21.8)
*IL-8* -251A carrier status	12.0	(-6.5, 28.2)	-	-
*GSTM1* null	9.0	(-18.9, 28.3)	9.6	(0.8, 19.0)
*GSTT1* null	0.9	(-8.2, 8.8)	4.3	(0.0, 12.4)
*CYP2E1* PstI/RsaI c2c2	1.8	(-2.0, 4.7)	-	-
*MTHFR* 677TT	10.0	(4.7, 16.4)	9.9	(-8.7, 26.5)
*MTHFR* 1298CC	-16.0	(-100, 28.7)	-	-
*p53* codon 72 Arg/Arg ^	-3.9	(-11.3, 0.34)	4.1	(-5.7, 15.2)
*E-cadherin *-160A carrier status	-9.2	(-26.0, 0.9)	18.5	(1.1, 33.4)

° PAR computed using meta-OR (wild-type homozigotes as reference) and the proportion of exposed cases (inverse-variance weighted mean derived from primary studies included in the meta-analyses);

§ confidence interval from Bonferroni inequalities [16];

^ OR using Arg/Pro and Pro/Pro individuals as reference.
